# Signal-Targeted Therapies and Resistance Mechanisms in Pancreatic Cancer: Future Developments Reside in Proteomics

**DOI:** 10.3390/cancers10060174

**Published:** 2018-06-01

**Authors:** Célia Cintas, Thibaut Douché, Nicole Therville, Silvia Arcucci, Fernanda Ramos-Delgado, Céline Basset, Benoît Thibault, Julie Guillermet-Guibert

**Affiliations:** 1 INSERM U1037, CRCT, Université Paul Sabatier, 31037 Toulouse, France; celia.cintas@inserm.fr (C.C.); thibaut.douche@pasteur.fr (T.D.); nicole.therville@inserm.fr (N.T.); silvia.arcucci@inserm.fr (S.A.); fernanda.ramos-delgado@inserm.fr (F.R.-D.); Basset.Celine@iuct-oncopole.fr (C.B.); benoit.thibault@inserm.fr (B.T.); 2 Laboratoire d’Excellence TouCAN, 31037 Toulouse, France; 3 Pathology Department, IUCT-O, Hôpitaux de Toulouse, 31037 Toulouse, France

**Keywords:** pancreatic cancer, proteomics, PI3K pathway, precision medicine, predictor of therapeutic response, integrated biology, proteo-genomics

## Abstract

For patients with metastatic pancreatic cancer that are not eligible for surgery, signal-targeted therapies have so far failed to significantly improve survival. These therapeutic options have been tested in phase II/III clinical trials mostly in combination with the reference treatment gemcitabine. Innovative therapies aim to annihilate oncogenic dependency, or to normalize the tumoural stroma to allow immune cells to function and/or re-vascularisation to occur. Large scale transcriptomic and genomic analysis revealed that pancreatic cancers display great heterogeneity but failed to clearly delineate specific oncogene dependency, besides oncogenic Kras. Beyond these approaches, proteomics appears to be an appropriate approach to classify signal dependency and to identify specific alterations at the targetable level. However, due to difficulties in sampling, proteomic data for this pathology are scarce. In this review, we will discuss the current state of clinical trials for targeted therapies against pancreatic cancer. We will then highlight the most recent proteomic data for pancreatic tumours and their metastasis, which could help to identify major oncogenic signalling dependencies, as well as provide future leads to explain why pancreatic tumours are intrinsically resistant to signal-targeted therapies. We will finally discuss how studies on phosphatidylinositol-3-kinase (PI3K) signalling, as the paradigmatic pro-tumoural signal downstream of oncogenic Kras in pancreatic cancer, would benefit from exploratory proteomics to increase the efficiency of targeted therapies.

## 1. Introduction

To date, surgical resection provides the best chance of a cure for patients with pancreatic cancer. However, this curative approach is only proposed in 15 to 20% of cases. Moreover, there is a high rate of relapse following tumour resection, with a greater than 50% chance of local recurrence after surgery. The 5-year overall survival rate in resected patients is 28% and the median survival time is 18 months [1]. This high rate of recurrence is associated with the presence of micrometastasis at the time of surgery. Hence, adjuvant chemotherapy is often prescribed and can significantly increase patient survival. A very recent meta-analysis of 14 articles showed that adjuvant treatment (chemotherapy or chemoradiotherapy) improves the survival of patients with pancreatic ductal adenocarcinoma (PDAC) at one year and three year [2]. In this context, the use of targeted therapies, including those targeting the phosphatidylinositol-3-kinase (PI3K)/Akt pathway, needs to be considered [3].

Indeed, in PDAC there are many altered signalling pathways downstream oncogenic Kras such as mitogen-activated protein kinase (MAPK) and PI3K pathways, but also Braf signalling, transforming growth factor (TGFβ), Notch and DNA repair pathways [4] and among them, the PI3K/Akt is one of the most critically affected. The lipid kinase PI3Kα was shown to drive initiation of pancreatic cancer downstream of the main driving oncogene of this cancer [5,6,7,8], oncogenic Kras, which is found to be mutated in more than 80% of pancreatic cancer patients.

For 80–85% of patients with non-operable pancreatic cancer with locoregional or distant metastases (mainly liver and lung) chemotherapy can be used to improve quality of life and survival by relieving symptoms of disease. Since 1997, gemcitabine monotherapy remains the standard palliative chemotherapy for patients with metastatic PDAC [9,10,11,12], given the many clinical failures to combine it with other agents [13]. Recently, the combination of gemcitabine and nab-paclitaxel was found to be superior to treatment with gemcitabine alone (8.5 months versus 6.7 months survival), but presented a higher toxicity [14,15]. nab-paclitaxel is an albumin-bound paclitaxel, with the addition of albumin possibly leading to improved pharmacokinetics, greater specificity of distribution within the tumour, higher intra-tumoural concentration and better efficacy. The first major advance in the palliative treatment of patients with pancreatic cancer occurred in 2011 using FOLFIRINOX, a combination chemotherapy combining 5-fluorouracil, folinic acid, irinotecan and oxaliplatin. It has become the standard treatment for metastatic PDAC in patients in good general condition. Its efficacy is superior to that of gemcitabine (mean overall survival of 11.1 months versus 6.7 months), but it produces more toxic side effects [16,17]. If the determination of the most efficient chemotherapy regimen is improving, how to select the most efficient targeted therapy for these patients remains an open question in the field.

In 2016 the American Society of Clinical Oncology (ASCO) published its recommendations for the management of potentially curable PDACs (grade I, II), locally advanced PDACs (grade III), and metastatic PDACs (grade IV) (www.asco.org/guidelineswiki). Their recommendations for therapeutic interventions, including FOLFORINOX, irradiation and/or gemcitabine in combination with nab-paclitaxel, are based on relevant articles published between 2004 and 2015. However, it remains necessary and urgent to discover new targets and associated therapeutics, as well as improve irradiation protocols and current therapeutic strategies, by determining the mechanisms of intrinsic pancreatic resistance to anti-tumour agents including targeted therapies. Targeted anti-cancer therapies are strategies that aim to block or annihilate cancer progression by specifically addressing some of their abnormalities, including at the protein level. Here, we will first analyse the state-of-the art clinical trials involving targeted therapies in pancreatic cancer and, in light of the latest studies on PDAC proteomes, discuss how analysis of protein levels or post-translational modifications by proteomics can aid in the design of efficient personalized therapies for pancreatic cancer patients.

**Figure 1:**
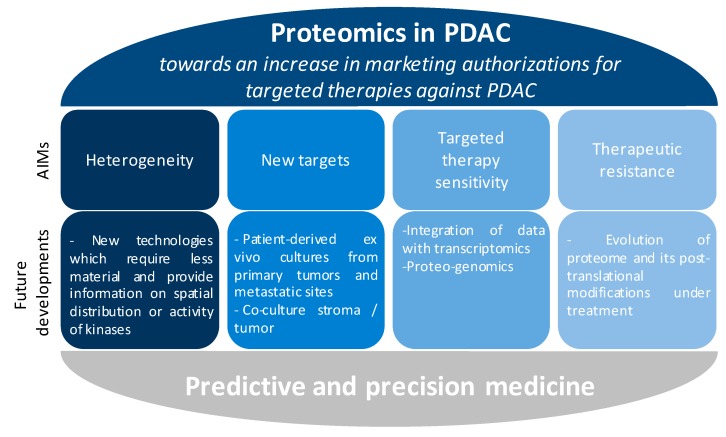
Using proteomics to improve the clinical care of patients with pancreatic cancer.

Proteomics (see Figure 1) is a powerful and promising tool for:

- the identification of early diagnostic and prognostic protein biomarkers;
- the identification of deregulated proteins and signalling pathways;
- the identification of protein biomarkers predictive of the response to treatment;
- the identification of early resistance mechanisms leading to the development of adapted combinatorial treatments.

It has now become clear that state-of-the art proteomics can be applied to PDAC research to help address these and other questions related to the clinical management of PDAC (Table 1). However, with the exception of studies into biomarkers, the proteomics data obtained for this pathology remains poor compared to data for other liquid or solid tumours.

### 1.1. Proteomics Is a Technological Tool Which Can Help to Improve the Clinical Management of Pancreatic Cancer

Proteomics is the study of all the proteins of an organism, a biological fluid, an organ, a cell or even a cellular compartment. This set of proteins is called the “proteome”. The latter is a dynamic and complex entity. The proteome contains a much larger number of proteins than the genome contains genes. In human cells, an estimated 22,000 genes can yield up to one million proteins [18]. Despite these approximations, it is considered that proteins represent about 60% of a cell. The large scale study of proteins grew dramatically during the 1990s, with the advent of mass spectrometers (in 2002 the Nobel Prize in Chemistry was awarded to John Fenn and Koichi Tanaka for developing a novel method for the mass spectrometric analysis of biological macromolecules) [19]. Mass spectrometry (MS) is an analytical method that ionizes chemical species and sorts the ions based on their mass-to-charge ratio; it aims to identify and separate molecules (proteins but also small molecules and drugs) with very good resolution and sensitivity. New methodologies now allow the qualitative and quantitative analysis of complex biological samples, which may contain thousands of proteins, some of which are present in small quantities [20,21]. Post-translational modifications such as phosphorylation and ubiquitination can be studied by these techniques with prior enrichment of the starting material (mostly with beads coated with anti-pTyr antibodies or anti-ubiquitin antibodies for studies of tyrosine phosphorylations or ubiquitinations, and immobilized metal affinity chromatography (IMAC) or titanium dioxide (TiO_2_) beads for serine, threonine, or tyrosine phosphorylations) [22,23]. Such studies are called phosphoproteomics and ubiquitin-omics, respectively. Post-translational modification can regulate the biological activity of a protein. Therefore, analyses of the post-translational modifications of proteins in a tumour could identify the Achille’s heal of each tumour, and lead to the identification of novel therapeutic targets [24].

**Figure 2:**
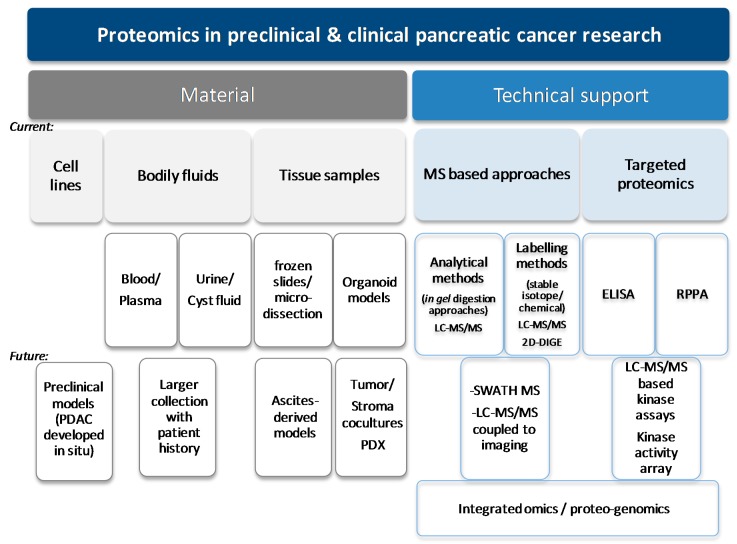
Major materials and technical tools used in preclinical pancreatic cancer research*.* RPPA: reverse phase protein array; ELISA: Enzyme-linked immunosorbent assay; LC-MS/MS: Liquid chromatography–tandem mass spectrometry; 2D-DIGE: two-dimensional differential gel electrophoresis; PDX: patient derived xenograft.

Proteomics provides insights into proteome-related changes in disease, such as observed changes in protein abundance, subcellular localization, post-translational modifications and cell signalling. Published proteomic results for pancreatic cancer are derived from the analysis of fluid (blood [25], plasma [26,27,28,29,30], serum [27,28,31,32,33,34,35,36,37,38,39,40,41], pancreatic juice [42] and cystic fluid [43]) and/or solid samples mostly microdissected beforehand [30,44,45], not from formalin-fixed-paraffin embedded (FFPE) tissue sections but from frozen sections [31,33,44,45,46,47], from healthy versus sick patients or patients at risk of developing pancreatic cancer (e.g., chronic pancreatitis (CP) [27,29,31,32,34,35,37,38,39,42]), from murine models of PDAC [30,33,48,49,50], as well as from conditioned medium [37,41,50,51,52] and established or experimental tumor-derived or patient-derived cell lines [25,27,30,37,48,50,51,52,53,54,55,56] (Figure 2).

Some articles report the quantification of total or phosphorylated protein amounts using reverse phase protein array (RPPA) or “reversed phase protein chips” [27,37,47] and enzyme-linked immunosorbent assay (ELISA), mostly for validation purposes [26,28,34] (Figure 2). Nevertheless, pancreatic cancer samples are mainly studied using higher throughput methods such as mass spectrometry or two-dimensional differential gel electrophoresis (2D-DIGE) [30,41,42,44,45,52].

Bottom-up (or shotgun) approaches, that is, the identification of proteins from the analysis of their peptide components, have led to increased proteome coverage (i.e., the identification and quantification of a maximal number of proteins from a mixture). Tryptic digestion followed by peptide fractionation, peptide separation through liquid chromatography, and on-line electrospray ionization coupled to tandem MS/MS orbitrap-based analyzers is the most common choice for in-depth proteomic analysis. Improvements in sample preparation, the sensitivity of mass analyzers and computational developments now allow researchers to quantify what is considered to be close to a full proteome (over 10,000 proteins expressed per cell) [57]. This level of protein identification and quantification is rarely described in studies involving PDAC samples [50]. In PDAC, due to the low amount of samples, key studies involve the utilization of more discriminatory techniques such as 2D-DIGE [45], that consists of labelling proteins with fluorescent probes prior to a 2D-electrophoresis separation according to their isoelectric point and molecular weight. Subsequently, spots can be excised from the gel, proteins digested with trypsin and peptides identified by matrix-assisted laser desorption/ionization time-of-flight (MALDI-TOF). Laser capture microdissection (LCM) has proven to be a beneficial preanalytical tissue-processing technique for isolating and selectively enriching discrete cellular populations from frozen tissue sections [30,44,45]. In PDAC, desmoplasia (tumour microenvironment) can pollute samples. Laser dissection can be used to discriminate epithelial cells from stroma and hence increase the chance of discovering biomarkers of early disease [30,44,45].

For quantitative proteomic approaches (as reviewed in [58]), differences in sample preparation, sample injection, and between each MS run can have profound effects on MS results. This is particularly true when attempting to quantify differential protein abundance between samples. This results in large numbers of replicate runs being required before any quantitative difference can be confidently identified. This is critical in all label-free MS experiments [57]. To overcome this, mass tags, such as stable isotope labelling with amino acids in cell culture (SILAC), isobaric tags for relative and absolute quantitation (iTRAQ), and chemical dimethyl labelling, can be incorporated into samples, typically labelling proteins or peptides, during tissue culture (SILAC) or sample lysis (superSILAC, iTRAQ and dimethyl labeling). The addition of these mass tags to one of the comparative groups allows multiple samples to be mixed and prepared in an identical manner while facilitating their separation within the mass spectrometer. Such labelling techniques allow the simultaneous investigation of multiple samples, facilitating accurate relative quantification, reducing bias, and increasing reproducibility [57] and are critical to reproducibly study post-translational modifications. These techniques are starting to be explored in PDAC [35,46,48]. Other strategies involve LC-MS/MS based [59] or protein array [60] technologies to measure and infer selected kinase activity (Figure 2). Although of easier use, targeted proteomics on protein expression and phosphorylation alterations most commonly found in cancer by techniques such as RPPA do not always give satisfactory answers due to their possible lower sensitivity and their narrowed analysis [48]. Mass spectrometry-based proteomic profiling of well-characterized cohorts of PDAC patients with detailed clinical information has the potential to provide effective stratification of PDAC patients and identify potential targets for novel treatments.

### 1.2. Genomic Characterization Has Failed to Identify Molecular Subtypes of Pancreatic Cancer to Guide the Choice of Targeted Therapies; Proteomics Appears to Be A Better Approach

**Figure 3:**
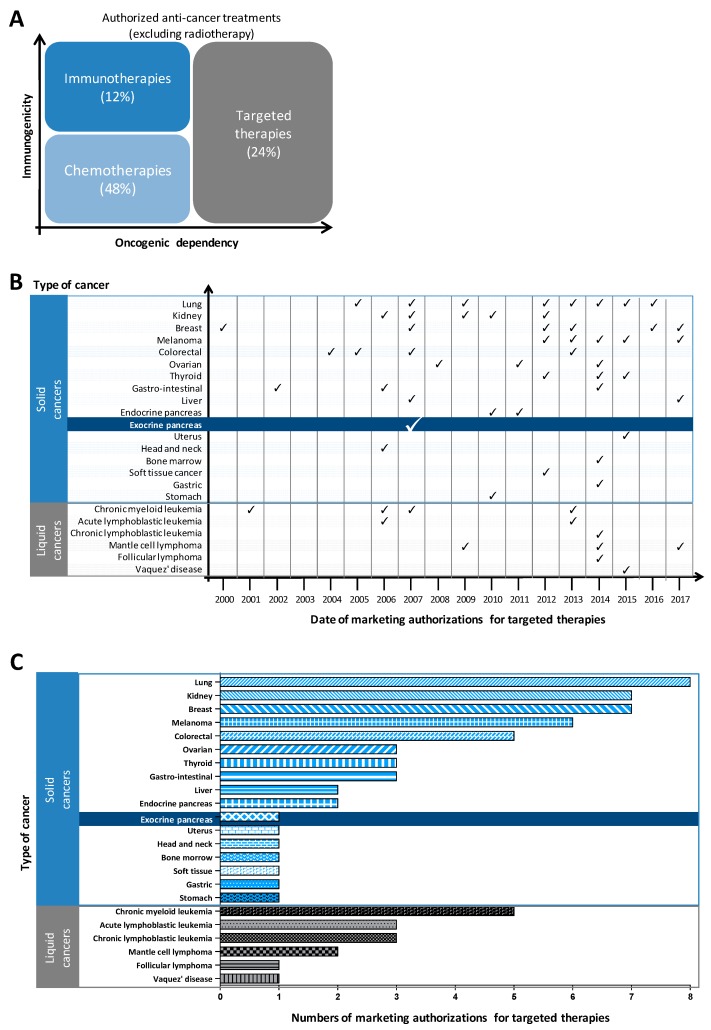
Evolution of the use of targeted therapies in cancers using France as an example. (**A**) Contribution in percentage of targeted therapies as compared to the therapeutic arsenal authorized for use in cancers (excluding radiotherapy). Distribution (**B**) and number (**C**) of marketing authorizations for targeted therapies. Adapted from INCa 2015 and completed with clinicaltrials.gouv.

Anti-cancer drugs fall into four broad categories based on their pharmacological action: conventional chemotherapy, radiopharmaceuticals, immunotherapies and inhibitors of (mostly) oncogenic mechanisms (but also of angiogenesis or immunomodulation) that include targeted therapies and hormone therapies (Figure 3A). Targeted anti-cancer therapies are strategies that aim to block the growth and/or spread of tumour cells by specifically addressing some of their abnormalities. Their main mode of action is an inhibition of the mechanisms of oncogenesis with a higher specificity towards cancer cells or their microenvironment. They are intracellular inhibitors (small chemical molecules such as protein or lipid kinase inhibitors) or extracellular inhibitors (biological drugs such as monoclonal antibodies to receptor tyrosine kinases (RTKs) or their ligands) (Table 2).

Targeted therapies are part of what is called “precision medicine”. This term refers to a medicine that is based on an in-depth knowledge of the biological mechanisms leading to the appearance and development of tumours. The use of these treatments is therefore guided, as far as possible, by the molecular characteristics of each patient’s tumour (for example: tumour differentiation, genetic alterations such as mutations/overexpression of oncogenes, loss of function of tumour suppressor genes, proteome). Most targeted therapies are currently used as a monotherapy (62% in France). The first targeted therapy was approved for use in France in 2000. This was trastuzumab, an antibody targeting the extracellular domain of the HER2 receptor, used in the treatment of human epidermal growth factor receptor 2 (HER2)-positive metastatic breast cancer as a monotherapy in patients that had already been treated with at least two chemotherapies for their metastatic disease. By the end of 2015, the French Cancer Health Institute INCa had identified 47 targeted therapies which had marketing authorization (MA) in France for the treatment of cancer.

Only one molecule, an inhibitor of the RTK epidermal growth factor receptor Epithelial Growth Factor Receptor (EGFR), has an indication in PDAC, used in combination with gemcitabine without any patient stratification (Figure 3B,C). So far, the efficiency of these molecules in PDAC remains modest, if compared to the spectacular action of targeted therapies in the first year of treatment in other aggressive solid tumours. In the successful cases, the choice of therapy is guided by oncogenic alterations, such as BRAF inhibitors in melanomas or EGFR inhibitors in lung cancers. Although overall survival (OS) and progression-free survival (PFS) improved slightly in PDAC patients treated with an EGFR inhibitor in combination with gemcitabine, there was no significant difference in the objective response (measurable response) as compared to placebo plus gemcitabine [61,62]. At present, in France, 38 clinical trials testing one or more anti-cancer molecules are in progress for pancreatic ductal cancer (data excluding neuroendocrine tumours), including 20 novel targeted therapies mostly used in combination with chemotherapy (Table 3). These studies were designed without the inclusion of any predictive markers. Targeted therapies against pancreatic microenvironment, which is thought to contribute to tumour aggressiveness, are not yet in phase I clinical trials [63].

In PDAC, the efficacy of 15 targeted therapies (mostly in combination with the chemotherapy gemcitabine) has been evaluated [64], the details of which are presented in Table 4. Despite patients displaying an initial anti-tumour response after treatment with these targeted therapies, as demonstrated by an ameliorated general state of the patients, resistance is induced very quickly. Besides, the poor general state of patients before starting their treatment prevents an increase in dosage required to reach a better target coverage and is an issue when testing a combination of several targeted therapies due to their increased toxicity. In the case of mTOR inhibitors, clinical trials led to decreased survival, possibly due to an acceleration of resistance mechanisms as explained below (paragraph 1.6). In general, it has been found that improvements to patient survival time are limited to a few weeks-months. Therefore, more effective drug combinations which prevent resistance while sparing general toxicity need to be developed.

Pancreatic cancer cells are mostly initially resistant (corresponding to a process called innate resistance). Moreover, adaptive resistance can also occur in pancreatic cancer. Whereby one or more tumour subpopulations with different characteristics compared to the sensitive cells could emerge allowing them to survive and continue to proliferate in the presence of pharmacological inhibition, leading to therapeutic failure [65,66]. Heterogeneity within a tumour is thus a critical component of resistance mechanisms [67]. Technological progress and large volumes of transcriptional, epigenetic and genetic data have led to a characterisation of the molecular identity of pancreatic cancer (mostly from resected tumours) [68,69,70,71]. However, in contrast to other cancers such as in lung cancer with the search of *EGFR* mutations at the genome level, these global approaches failed to identify simple therapeutic strategies based on a stratification of PDAC patients. Recent integrated omics approaches for all cancers highlight that the search for genetic or genomic alterations is not sufficient to predict which patients will benefit from targeted therapies [69,72]. In contrast, proteomic approaches appear better than transcriptomics or genomics at predicting sensitivity to a targeted therapy (for example PI3K inhibitors) because they reflect the real activity of the targets in tumors or in metastases [72,73]. It is all the more important in PDAC where the genomic and transcriptomic heterogeneity does not lead to obvious molecular classifications. This suggest that proteomic methods should be used to investigate resistance to treatment in pancreatic cancer patients, and moreover predict which therapy will be most efficient (Figure 1) [73]. Recent pioneering data, which need to be complemented by wider studies, also argue for the development of such unbiased exploratory strategies in pancreatic cancer.

### 1.3. Proteomic Profiling of PDAC Tissues

Proteomic studies give insights into metastatic PDAC biology, leading to the discovery of novel targets. Oncogenic Kras is altered in more than 80% of all PDAC patients. In PDAC, preneoplastic lesions (pancreatic intraepithelial neoplasia (PanIN), mucinous cystic neoplasm (MCM) and intra-ductal papillary mucinous neoplasm (IPMN)) progressively evolve towards at first in situ and then infiltrating metastatic adenocarcinoma, histologically defined as differentiated, moderately differentiated or dedifferentiated. Molecular classifications based on transcriptomics and genomics data have now been developed, including so called squamous, pancreatic progenitor, immunogenic and aberrantly differentiated subtypes [68,69,70,71]. The majority of proteomic studies performed to identify biomarkers of pancreatic cancer do not discriminate the different grades of the tumours studied or refer to the new molecular classification. This is due to the relatively low number of patients used in these studies (maximum 36 cell lines [54], 46 tumour samples [44], 173 plasma samples [25], serum samples [37] or 24 cystic fluid samples [43]), with sub-classification rendering a statistical approach irrelevant. However, 2D-DIGE technology performed on microdissections of human pancreatic tumours identified that the calcium-binding protein S100A6 was upregulated in moderately or poorly differentiated tumours [44], and was found to regulate epithelial-mesenchymal transition via an activation of β-catenin [74]. In 2009, Sitek and collaborators identified 86 differentially regulated proteins involved in pancreatic tumour progression using only microdissections of 37 single lesions of different grades of PanIN (PanIN is a precursor lesion of PDAC) from 9 patients, compared against PDAC cells and normal ductal cells [45]. These results are complemented by proteomic analysis performed by Dufresne et al., involving 2D-DIGE of microdissected murine PanIN cells and plasma samples from genetically engineered mouse models of the different precancerous stages [30]. The identification of peptide signatures specific to each type of precancerous lesion would thus make it possible to discriminate them from normal pancreatic tissue and could potentially lead to the discovery of both biomarkers and targets.

Independently of PDAC molecular classification, one study proposes the classification of pancreatic cancer cell lines based on the level and site of tyrosine phosphorylation (pTyr) of RTKs, potentially identifying three groups of cell lines classified as “low pTyr”, “RTK enriched pTyr” and “mixed pTyr” (on BAIAP2 Y337, PKP3 Y84, PKP2 Y166, CTNND1 Y174, CTNND1 Y 904) [54]. All sub-types presented a high level of tyrosine phosphorylation of RON, EPHA2, MET and ERBB2. “RTK enriched pTyr” cell lines presented high levels of tyrosine-phosphorylated EGFR, MET, RON, EPHA4, EPHB2/3/4, DDR2 as compared to the two other subtypes. These data indicate that a combination of RTKs are usually activated in pancreatic cancer, suggesting that single agent strategies towards RTKs are likely to be inefficient. If this classification could be performed on patient biopsies, it could guide the choice of treatment (including combinative strategies). A diagnostic tool based on proteomic profiling of easy-to-access liquid biopsies could be a clinically-feasible approach, as recent data show that the surface proteome of circulating exosomes provides unique opportunities to analyse the heterogeneity of metastatic pancreatic cancer [25].

A large scale proteomic analysis of a cell line with stemness properties (a subclone of Panc-1 cells characterized by increased expression of ceruplasmin, galectin-3 and myristoylated alanine-rich C-kinase substrate MARCKS) and its secretome revealed the importance of fatty acid synthesis and mevalonate pathways, as well as glycolysis, through the regulation of fatty acid synthase FASN and acetyl-CoA acetyltransferase2 ACAT2 protein expression, in this subtype of cells. Results obtained from this study could accelerate the development of novel therapeutic strategies targeting pancreatic cancer stem cells [51,53]. Using lectin microarray and nano LC-MS/MS, several glycoproteins were found to be enriched (e.g., cytokeratin 8 or ICAM1) or down regulated (e.g., aminopeptidase N) in this population of cells [75]. Stem cells may be the origin of tumour relapse in patients undergoing chemotherapy, therefore the discovery of specific targets for this pool of cells from patient-derived organoids could improve the current treatments. Indeed, large scale proteomics data on patient-derived organoids or xenografts (PDX) are lacking (Table 1); these data would provide a proof-of-concept for the use of proteomics in precision medicine (Figure 1).

### 1.4. Proteomic Approaches to Search for New Biomarkers of Early Disease

Early detection of pancreatic cancer is key for improving patient survival. Recent review articles discuss the use of several proteomic techniques for the early diagnosis of PDAC [76,77,78]. Currently, clinically used tumour markers lack sensitivity and specificity. For example, the carbohydrate antigen marker CA-19-9 (secreted by exocrine cells and tumours) present in the serum makes it possible to estimate pancreatic tumour progression during treatment [79]. However, the presence of this marker is not sufficient to diagnose pancreatic cancer, due to its lack of specificity (a high concentration is also found in the sera of patients with acute and chronic pancreatitis, hepatitis and biliary obstruction).

After the identification of potential protein biomarkers by large scale approaches, in the long term, the development of targeted proteomic technologies, such as selected reaction monitoring (SRM) also called multiple reaction monitoring (MRM) (using other MS apparatus such as triple quadrupole mass spectrometers), which can provide accurate and reproducible measurements of biomarkers of interest may be critical for the clinical use of the identified protein biomarkers [57]. To confirm the identity of a protein a match to at least two proteotypic peptides (peptides that are most likely to be identified by current MS-based methods, unique to this protein, without missing cleavage sites and not susceptible to post-translational modifications) is required. In the cancer proteome, aberrant proteins are present, which are absent from protein databases. This can also explain why the identification of cancer specific peptides is difficult, especially for this very heterogeneous type of cancer.

Numerous studies report new specific protein biomarkers for the diagnosis of pancreatic cancer identified from pancreas tissue lysate or liquid biopsies (such as secretome, but mostly plasma/serum) by proteomics [31,32,36,37,38,39,40,41,46,52,80]. Some biomarkers have been characterized and evaluated alone or in combination with CA19-9 for their ability to prediagnose pancreatic cancer [34]. For example, Park et al. established recently, from a large-cohort study of 500 patient sera, a panel of two proteins, apolipoprotein A4 (APOA4) and tissue inhibitor of metalloproteinase-1 (TIMP1), that in combination with CA 19-9 detection, were able to discriminate pancreatitis from early PDAC [38]. Preneoplastic samples are difficult to obtain due to the late diagnosis of this disease, nevertheless a recent analysis of 25 human PanIN organoids has led to a major step forward. Thrombospondin 2 detected in plasma either alone or in combination with CA19-9 was validated (98% combination specificity, 87% sensitivity) to distinguish all stages of the disease (resectable I and II, locally advanced III and metastatic IV), and in particular was able to discriminate resectable stage I cancer as readily as stage III/IV PDAC tumors [81]. Studies of other preneoplastic lesion such as IPMN and other cystic precursor lesions of the pancreas identify the proteins TIMP1 in plasma, mucin-5AC, mucin-2, cyst-fluid carcinoembryonic antigen and prostate stem-cell antigen in cyst fluids as potential protein biomarkers [29,43]. These studies should lead to further insights regarding the early diagnosis of pancreatic cancer. Identification using iTRAQ and validation using MRM of thrombospondin-1 (TSP-1) as a protein biomarker in patients at risk of developing pancreatic cancer, such as those with neo-onset diabetes, show that this condition impacts positively the diagnostic performance of CA19-9 [35]. These advances in early diagnostics are expected to lead to an increase in the efficiency of targeted therapies.

Despite the successful identification of biomarkers in preclinical or early phase clinical proteomic studies, thus far no proteomics-based discovery is used in clinical practice to diagnose PDAC. Honda et al., recently developed an antibody-based assay (ELISA) to measure circulating apolipoprotein A2 (APOA2) isoforms APOA2-ATQ/AT (C-terminal truncations of the apoAII homo-dimer) for the early detection of pancreatic cancer. However, clinical validation on 151 cases of stage I/II pancreatic cancer did not allow the early stages of pancreatic cancer to be statistically distinguished from healthy controls. Its clinical use should be reassessed in patients at high risk of pancreatic malignancy [28,34,35]. The definition of high risk patients remains difficult in PDAC. No single protein biomarker has a high enough discriminatory power to diagnose PDAC, therefore the development of a panel of multiple protein or glycoproteins biomarkers [82], which are more sensitive and specific, is essential for the expansion of routine clinical tools.

As is the case for most of these studies, replication of the results in larger cohorts of patients is necessary to form conclusions regarding the use of individual markers. Alternative approaches, such as the sequential window acquisition of all theoretical mass spectra (SWATH-MS) methodology which was developed recently [83], based on data-independent acquisition, can provide large coverage with a high degree of reproducibility (Table 1). Indeed, SWATH-MS allows a complete and permanent recording of all fragment ions of the detectable peptide precursors present in a biological sample. It thus combines the advantages of shotgun (high throughput) with those of SRM (high reproducibility and consistency). However, this technology has not yet been largely applied to PDAC patient samples.

### 1.5. Proteomic Approaches to Search for New Biomarkers of Predictive Response

Global bottom-up approaches to analyse the proteome and post-translationally modified proteomes (phosphorylation, glycosylation, ubiquitination) are scarcely used in clinical PDAC. Indeed, proteomics can be used as an adapted tool to predict the response to targeted therapies. Studying inter-patient tumour heterogeneity with quantitative MS-based high throughput proteomics and phosphoproteomics (as performed for 14 resected head PDAC patients) allows the identification of commonly activated signals such as Akt activation [46]. Proteomics can also analyse the changes in signalling pathways during treatment course of patients [67]. Using a quantitative proteomic approach (using first SILAC for protein labelling, then an enrichment in tyrosine phosphorylated peptides), Kim and collaborators revealed the heterogeneity of three metastatic sites of pancreatic cancer (liver, lung and peritoneum) by creating three cell lines from each organ of the same patient [55]. This heterogeneity is characterized by changes in the expression of the entire proteome as well as changes in the activity of tyrosine kinases, such as Axl tyrosine kinase. These data further demonstrate that neoplastic cells developing in different organs in the same PDAC patient display differential sensitivity to targeted therapies. So far, these properties have not been taken into account in clinical trials, partly explaining their relative inefficiency (Table 3 and Table 4). In contrast, in a large scale genomic and genetic analysis, genetic alterations in metastatic sites were found to be maintained as compared to the primary site of tumourigenesis [67]. Thus, the latest proteo-genomics data highlight the importance of a personalized combination of therapeutics targeting all the subclonal features of metastases, using proteomics to guide the therapeutic choice.

Proteomics has been found to be a powerful tool in the study of tumour cell/microenvironment cell signalling interactions and modifications. For example, Jorgensen’s team has recently demonstrated using murine Kras-mutated pancreatic cancer cells that oncogenic Kras signalling in tumour cells not only activates a cell-autonomous signalling network, but that it also activates the non-autonomous oncogenic signalling of stromal cells through a selective modification of their proteomes and their post-translationnal proteomes. Conversely, stromal cells can in turn modify and amplify oncogenic signalling increasing the number of protein signalling nodes and activated kinases involved in the survival of the same or other tumour cells. The total proteome or the phosphorylated proteome is different when cells are co-cultured, and lead to the identification of another oncogene dependency (here, insulin growth factor receptor 1 IGFR1/Axl). Thus, oncogenic signalling is no longer limited only to tumour cells but to the entire tumour compartment [50]. The temporal unbiaised exploration of extracellular communication signal would also help to design efficient signal targeted therapies. Indeed, the extracellular matrix found in abundance in desmoplasia is an important area to explore, using, for example, SWATH-MS technologies as developed for breast cancer [84].

Hence, besides genetic and genomic alterations, prediction of sensitivity to targeted therapies is influenced by heterotypic tumour-stroma signalling. The only way to assess this sensitivity appears to be by proteomic analysis of patient-derived samples, including all the cellular partners at stake in the primary tumour and the metastatic niche (Figure 1). This will require a proper analysis of post-translationally modified proteins in each cell compartment when cultured alone of in combination with their environment. Cross-comparison with heterotypic signalling in tumors from a different organ could pave the way to explain the intrinsic resistance to targeted therpies of pancreatic cancer.

### 1.6. Proteomic Approaches to Identify Resistance Mechanisms-Towards an Evolution in Precision Medicine

Global studies to understand the adaptive responses to targeted therapies in pancreatic cancer are starting to be published. Adaptive and reversible resistance to inhibition of cancer-driving mutated Kras in pancreatic cancer cells has been found to involve the tyrosine phosphorylation of focal adhesion pathway components, while strikingly no significant mutational or transcriptional changes were observed [48]. Alteration of other post-translational modifications other than tyrosine phosphorylation was not analysed. Large scale proteomics analysing the temporal effects of paclitaxel on pancreatic cancer cells, highlighted the role of proteins involved in mitochondrial function, survival (PI3K pathway) and cell cycle arrest in a key resistance mechanism [56]. Proteomics has identified that several negative feedback mechanisms are relieved upon mTORC1 inhibition, thus explaining the disappointing results from clinical trials on this target [38,39,57,59]. In other cancer settings, Hsu and colleagues have shown, for example, through global phosphoproteome analysis after SILAC labelling of cells (a method of labelling cells which allows a very precise differential quantitative approach) and enrichment in serine-, tyrosine- and threonine- phosphorylated peptides that the mTORC1 complex is able to inhibit and degrade insulin and IGF-1 receptors through phosphorylation of the adapter protein growth factor bound protein 10 (Grb10) [85,86]. It is now generally agreed that to target the PI3K/Akt/mTOR pathway, which is hyperactivated in 50% of all PDAC patients and associated with poor prognosis [6], hitting upstream PI3K is the best strategy to prevent positive feedback due to mTOR (mTORC1) inhibition. Whilst some mechanisms of resistance to PI3K inhibitors have been identified such as mitogen-activated protein kinase kinase activation also known as MEK [87,88], other mechanisms of resistance remain elusive in pancreatic cancer. Integrating different types of omics approaches to identify such mechanisms could increase the efficiency of these innovative targeted therapies towards driving signalling pathways [3,72].

## 2. Conclusions/Discussion/Perspectives

It is challenging to perform in-depth and large-scale clinical studies on PDAC. The sparsity of samples and the difficulty in defining the population at risk renders this task even more difficult. However, recent technological developments have led to advances both at fundamental and clinical research stages. Besides a better understanding of the oncogenic dependency of PDAC [89], the current development of MS-based methods, coupled to imaging [33,49], which need less material and are more quantitative [90] and of non-MS based robust targeted proteomic approaches [91], together with improvements to patient-derived ex vivo cultures to better mimic each patient’s situation will be instrumental in improving the management of patients with pancreatic cancer. Proteomic profiling of metastatic tumour cells will also be necessary (Table 1). Finally, integration of MS-based proteomic profiling with other state-of-the art technologies (such as next generation sequencing) will help to define driver proteins and to identify therapeutic targets. Integration of these data in a so called proteo-genomics approach is grabbing tremendous attention and has already been applied to other cancers [92]. Mostly used to provide protein-level evidence of gene expression and to help refine gene models, proteogenomics also includes computational strategies for building and using customized protein sequence databases. The later information are used to help identify novel peptides (not present in reference protein sequence databases) from mass spectrometry–based proteomic data [92]. These novel computational methods will be critical to achieve full personalized management of PDAC. Again, defining the best culture conditions, using conditions which mimic patient heterogeneity while maintaining the signalling pathways highly specific to the organ niche [50] is key to this success (Figure 1).

Cancers, such as pancreatic cancer, with complex molecular pathways are more refractory than those with less complex pathways [93]. Any improvement in the knowledge of this complexity will increase the efficiency of targeted-therapies in PDAC patients. This will only be possible first if we analyse in better detail cell-cell communication in simpler systems, while maintaining at best the conditions found in tumours or metastatic sites.

From reviewing the literature, it is clear that key questions still need to be answered (Table 1). Increased access to material from PDAC patients (via organoid culture), technologies adapted to the discovery of biomarkers such as SWATH technology and newly developed spatially resolved proteomic techniques will strive towards these aims. A few key questions are:

- Can we use proteomics to detect PDAC earlier? Are earlier detected tumours more sensitive to targeted therapies towards PDAC oncogenic dependency (e.g., PI3K)? Will this knowledge increase life expectancy of PDAC patients?
- Can we use proteomics to refine the current (epi)-genetic and genomic characterization of PDAC to better stratify patients? Sampling patients with PDAC is difficult. Can we develop and adapt methodological work flows to aid patient sampling? These workflows should also incorporate a better understanding of the metastatic disease.
- Can we use proteomics to identify new targets (extracellular, membrane or intracellular) which take into account tumour-stroma heterotypic signalling? Is this signal different in each tumoral niche?
- Can we use proteomics to understand, at a targetable protein level/modification (phosphorylation, ubiquitination), the specific resistance of PDAC patients to targeted therapies?

Early (or at least earlier) diagnosis using proteomics is a growing field with promising leads, but to be clinically validated larger cohorts of at-risk patients are needed. New leads in terms of oncogenic dependency have been discovered, such as the importance of PI3Kα in driving Kras-induced pancreatic cancerogenesis [5,8]. Clinical trials targeting such enzymes are on-going (Table 3 and Table 5). However, there is a lack of preclinical data to identify patients that are more sensitive to PI3K inhibitors due to an absence of commonly found alterations of the pathway (such as the loss of PTEN expression and the mutation of *PIK3CA* gene encoding PI3Kα) [3]. Similarly, adaptive resistance to such therapies cannot be anticipated. This increase in knowledge should be accompanied with efforts to adapt proteomic technologies to the limited amount of cells which can be accessed in PDAC patients [94].

In conclusion, the next challenge for PDAC proteomics will be to increase our knowledge so that patients can be stratified according to each innovative therapeutic approach (Figure 1). A more comprehensive knowledge of the resistance mechanisms induced by targeted therapies, observed at the level of the target protein, will allow researchers and clinicians to develop effective therapeutic strategies adapted to each target/oncogenic pathway in each patient-specific environment. Thus abolishing, preventing or delaying the appearance of resistance, which may ameliorate the survival curve of these patients.

## Figures and Tables

**Table 1:** Open questions in PDAC research that could be addressed by applying proteomics to a large range of biological samples. CTC: circulating tumoral cell; PDX: patient-derived xenografts.

Source	Details of Source	PDAC Clinical Application	PDAC Tumoural Biology	Actual Limitations/*Developments*
**Biopsies (fine needle under echo-endoscopy)**	Diagnostic Active surveillance in patients at risk (chronic pancreatitis, mucinous lesions, hereditary)	Diagnostic markers Prognostic markers Therapeutic options Markers of therapeutic response	Heterogeneity Molecular subtyping Stroma New targets Resistance to drugs	Poor cellularity *Development of organoids or PDX*
**Metastasis**	Loco-regional metastasis (peritoneum, ascites) Distant metastasis (lung, liver)	Diagnostic markers Prognostic markers Therapeutic options Markers of therapeutic response	Metastatic niche Molecular subtyping [55] Stroma New targets Resistance to drugs	Limited sampling
**Resection**	Normal adjacent tissue Tumour Desmoplastic reaction	Diagnostic markers [47] Prognostic markers Therapeutic options Markers of therapeutic response	Heterogeneity [31,44,45,46,47,52] Molecular subtyping Stroma New targets [46] Resistance to drugs	Limited to 15–20% of all PDAC patients-do not represent the most aggressive patients *Development of MS coupled to imaging*
**Body fluids**	Blood, blood fractions (serum, plasma, exosomes, CTCs, etc...) Urine Ascites	Diagnostic markers [26,27,28,29,32,34,35,36,37,38,39,40,41,42,43] Prognostic markers Therapeutic options Markers of therapeutic response	New targets Resistance to drugs	*Selection of patients based on circulating DNA* *Development of ascites-based PDX as an easy access to metastatic cells in their environment (e.g., immune cells)*
**Cell lines/in situ experimental PDAC**		Not applicable	Heterogeneity [30,33] Metastatic niche Molecular subtyping [54] Stroma [50] New targets Resistance to drugs [48,56] Secretions [50]	Do not fully represent the heterogeneity of PDAC *Study of heterotypic communication between stromal and cancer cells*
**Conditioned medium**		Not applicable	Secretions [25,37,41,51,52] Metastatic niche Molecular subtyping Stroma New targets Resistance to drugs	*Study of heterotypic communication between stromal and cancer cells*

**Table 2:** List of the 47 anti-cancer targeted therapies authorized in France. Adapted from INCa data and completed with Vidal.fr, updated in January 2018.

Intracellular Inhibitors		Extracellular Inhibitors	
Inhibitors of Protein Kinase(s)		Ab Directed against RTK(s)	
Name	Target(s)	Name	Target(s)
Afatinib	EGFR	Cetuximab	Ab anti-EGFR
Axitinib	VEGFR	Panitumumab	Ab anti-EGFR
Osimertinib	EGFR	Pertuzumab	Ab anti-HER2
Bosutinib	Bcr-Abl, Src	Ramucirumab	Ab anti-VEGF
Cabozantinib	MET, AXL, VEGFR, GAS6, RET, ROS1, FLT3, Tie2	Trastuzumab	Ab anti-HER2
Ceritinib	ALK	Trastuzumab emtansine	Ab anti-HER2
Cobimetinib	MEK	**Ab directed against ligand(s)**	
Crizotinib	ALK and MET	Aflibercept	Ab anti-VEGF
Dabrafenib	RAF	Bevabizumab	Ab anti-VEGF
Dasatinib	Bcr-Abl, Src	Denosumab	Ab anti-RANKL
Erlotinib	EGFR		
Everolimus	mTOR		
Gefitinib	EGFR		
Ibrutinib	BTK		
Idelalisib	p110δ (PI3K)		
Imatinib	Bcr-Abl, c-Kit, DDR1/2, CSF-1R, PDGFR		
Lapatinib	EGFR, ErbB2		
Lenvatinib	VEGFR, FGFR, PDGFR		
Nilotinib	Bcr-Abl		
Nintedanib	PDGFR, FGFR, VEGFR, FLT3, Lck, Lyn, Src		
Olaparib	PARP		
Osimertinib	EGFR		
Palbociclib	CDK4/6		
Pazopanib	VEGFR, c-Kit, PDGFR		
Ponatinib	Bcr-Abl		
Regorafenib	VEGFR, c-Kit, PDGFR		
Ribociclib	Cyclin D1/CDK4, CDK6		
Ruxolitinib	JAK1/2		
Sonidegib	SMO		
Sorafenib	RAF, VEGFR, FGFR, c-Kit, PDGFR		
Sunitinib	VEGFR, c-Kit, c-Kit, CSF-1R, RET, PDGFR		
Temsirolimus	mTOR		
Tivozanib	VEGF		
Trametinib	MEK1/2		
Vandetanib	VEGFR, EGFR, RET		
Venetoclax	Bcl2		
Vemurafenib	ERK, BRAF		
Vismodegib	SMO		

**Table 3:** Ongoing clinical trials for pancreatic cancer in France*.* In grey: clinical trials combining a targeted therapy with a chemotherapy. In blue: clinical trials using a targeted therapy only. Adapted from clinical.gouv.fr, updated in August 2017.

Name of the Study	Molecule Tested	Type of Therapy	Type of Drug	Phase	Pathologies
D081FC00001-POLO	Olaparib vs. placebo	Targeted therapy	Inhibitor of PARP	III	Metastatic PDAC with BRCA mutation
SIRINOX	Oxaliplatin + Irinotecan	Chemotherapy	Platinum salts, DNA topoisomerase I inhibitor	I	Digestive adenocarcinoma (pancreas, oesophagus, stomach, small intestine and biliary tract)
PRODIGE 29	FOLFIRINOX vs. Gemcitabine	Chemotherapy	Anti-metabolite, DNA topoisomerase I inhibitor, Platinum salts	III	Locally advanced PDAC
PAMELA-70	FOLFIRINOX	Chemotherapy	Anti-metabolite, DNA topoisomerase I inhibitor, Platinum salts	II	Metastatic PDAC
RC48	Adoptive transfer of allogeneic lymphocyte cells with natural cytotoxic activity + Cetuximab	Cellular therapy	Antibody anti-EGFR	I/II	Hepatic metastasis of PDAC, colorectal or small intestine cancer
PRODIGE 24 − ACCORD 24	Gemcitabine vs. FOLFIRINOX	Chemotherapy	Anti-metabolite, DNA topoisomerase I inhibitor, Platinum salts	III	PDAC
FIRGEMAX	Nab-paclitaxel + Gemcitabine vs. Nab-paclitaxel + Gemcitabine plus FOLFIRI3	Chemotherapy	Anti-metabolite, DNA topoisomerase I inhibitor, Platinum salts	II	Metastatic PDAC
PANOPTIMOX	FOLFIRINOX +/− LV5FU2 vs FOLFIRINOX +/− FIRGEM	Chemotherapy	Anti-metabolite, DNA topoisomerase I inhibitor, Platinum salts	II	Metastatic PDAC
MOAnab1	Gemcitabine + Nab-paclitaxel	Chemotherapy	Anti-metabolite	I	Metastatic PDAC
GABRINOX	Gemcitabine + Nab-paclitaxel followed by FOLFIRINOX	Chemotherapy	Anti-metabolite	I	Metastatic PDAC
JANUS-2	Ruxolitinib + Capecitabin	Targeted therapy + Chemotherapy	Inhibitor of Janus kinase (JAK) + Anti-metabolite	III	Locally advanced or metastatic PDAC
CMEK162X2111	MEK162 + Ganitumab	Targeted therapies	MEK inhibitor + antibody anti-IGF1R	I/II	Metastatic PDAC, colorectal adenocarcinoma and melanoma
AFUGEM	ABI-007 + Gemcitabine vs. ABI-007 + LV5FU2	Chemotherapy	Anti-metabolite	II	Metastatic PDAC
H9H-MC-JBAJ	Gemcitabine + LY2157299	Targeted therapy + Chemotherapy	TGFβR inhibitor + Anti-metabolite	I/II	Locally advanced or metastatic PDAC
2009-011992-61	Gemcitabine + AS703026	Targeted therapy + Chemotherapy	MEK inhibitor + Anti-metabolite	II	Metastatic PDAC
NEOPAC/IPC 2011-002	Neoadjuvant Gemcitabine + Oxaliplatin and adjuvant Gemcitabine vs. adjuvant Gemcitabine	Chemotherapy	Anti-metabolite +/− Platinum salts	III	PDAC (head of the pancreas)
CAOU6	Gemcitabine +/− ABI-007	Chemotherapy	Anti-metabolite	III	Metastatic PDAC
TherGAP	Anti-tumoural complex CYL-02	Gene therapy	Enzymatic, Metabolic	I	PDAC
ESPAC-4	Gemcitabine +/− Capecitabin	Chemotherapy	Anti-metabolite	III	Resectable PDAC
CO-101-001	Gemcitabine + CO-1.01	Chemotherapy	Anti-metabolite	II	Metastatic PDAC
GATE 1	Gemcitabine + Trastuzumab + Erlotinib	Targeted therapies + Chemotherapy	HER2 inhibitor, mTOR inhibitor, Anti-metabolite	II	Metastatic PDAC
ASTELLAS 200800	Gemcitabine + AGS-1C4D4	Targeted therapy + Chemotherapy	Antibody anti-PSCA + Anti-metabolite	II	Metastatic PDAC
AB SCIENCE AB07012	Gemcitabine +/− Masitinib	Targeted therapy + Chemotherapy	Tyrosine kinase inhibitor + Anti-metabolite	III	Locally advanced or metastatic PDAC
THERAPY	Cetuximab + Trastuzumab	Targeted therapies + Chemotherapy	HER2 and EGFR inhibitors	I/II	Metastatic PDAC
PANTER	Efavirenz	Targeted therapy	Inhibitor of non-nucleoside reverse transcriptase (NNRTI)	II	PDAC
GERCOR LAP 07 D07-1	Gemcitabine +/− Erlotinib followed by Gemcitabine or chemoradiotherapy with Capecitabin	Targeted therapy + Chemotherapy +/− Chemoradiotherapy	EGFR inhibitor + Anti-metabolite	III	Locally advanced PDAC
SciClone SCI-RP-Pan-P2-001	Gemcitabine +/−RP101	Targeted therapy + Chemotherapy	Hsp27 inhibitor + Anti-metabolite	II	Unresectable, locally advanced or metastatic PDAC
Hoffmann-La Roche BO21129	Erlotinib	Targeted therapy	EGFR inhibitor	II	Locally advanced PDAC
Pharmexa PRIMOVAX	Gemcitabine + GV001 vs. Gemcitabine	Targeted therapy + Chemotherapy	Stimulator of LT CD8 + Anti-metabolite	III	PDAC
Hoffmann-La Roche BO21128	Gemcitabine + Erlotinib	Targeted therapy + Chemotherapy	EGFR inhibitor + Anti-metabolite	II	Metastatic PDAC
Sanofi-Aventis EFC10203	tegafur (a prodrug of 5FU) + gimeracil (5-chloro-2,4 dihydropyridine, CDHP + oteracil (potassium oxonate, Oxo) vs. 5-FU	Chemotherapy	Anti-metabolite	III	Metastatic PDAC
Pfizer A4061028	Gemcitabine +/−AG-013736 (Axitinib)	Targeted therapy + Chemotherapy	VEGFR inhibitor + Anti-metabolite	III	Locally advanced or metastatic unresectable PDAC
Sanofi-Aventis EFC10547	Gemcitabine + Aflibercept	Targeted therapy + Chemotherapy	Antibody anti-VEGF1/2 + Gemcitabine	III	Metastatic PDAC
CAPERGEM	Gemcitabine + Capecitabin + Erlotinib	Targeted therapy + Chemotherapy	EGFR inhibitor + Anti-metabolites	I	Advanced PDAC
ACCORD 11 PRODIGE 4	Gemcitabine vs. FOLFIRINOX	Chemotherapy	Anti-metabolite, DNA topoisomerase I inhibitor, Platinum salts	III	Metastatic PDAC
ACCORD 09	Radiotherapy (RT) + Docetaxel + 5-FU or RT + Docetaxel and Cisplatin	Chemoradiotherapy	Radiotherapy, Alkylating agent, Anti-metabolite, Platinum salts	II	PDAC
Phase 1-2 (RECF0016)	Radiotherapy + Irinotecan	Chemoradiotherapy	DNA topoisomerase I inhibitor	I/II	Locally advanced PDAC
BAYPAN	Gemcitabine +/− Sorafenib	Targeted therapy + Chemotherapy	C-Raf and B-Raf inhibitor + Anti-metabolite	III	Locally advanced or metastatic PDAC

**Table 4:** Outcome of initial clinical trials for targeted therapies in pancreatic cancer*.* Some of the targeted therapies listed here did not have a high selectivity towards their targets [10,11,12].

Target	Therapy	Number of Patients	Mean Survival (Months) (Treatment Versus Chemo Only)
**Telomerase**	Gemcitabine + GV1001	1062	8.4 vs. 6.9
**VEGF**	Gemcitabine + Bevacizumab	602	5.7 vs. 6.0
**Kras**	Gemcitabine + Tipifarnib	688	6.3 vs. 6.0
**EGFR**	Gemcitabine + Cetuximab	766	6.5 vs. 6.0
	Gemcitabine + Erlotinib	569	6.24 vs. 5.91
**ErbB2**	Trastuzumab	44	4.6 vs. 5.4
**Gastrin**	Gastrazol + 5-FU	98	3.6 vs. 4.2
**mTOR**	Gemcitabine + Everolimus	29	4.5 vs. 6.5
**PI3K/PLK**	Gemcitabine + Rigosertib	106	6.1 vs. 6.4
**Sonic Hedgehog**	Gemcitabine + Vismodegib	106	6.9 vs. 6.1
**Notch3**	Gemcitabine + IPI-929	122	Not tolerated
**IGF1-R**	Gemcitabine + Ganitumab	800	7.0 vs. 7.2
**MMP**	Gemcitabine + Matrimastat	239	5.4 vs. 5.4
**JAK/STAT**	Ruxolitinib + Capecitabin	127	4.5 vs. 4.2
**α-secretase**	RO4929097 (no Gemcitabine arm)	18	4.1
**MEK1/ERK_1/2_**	Selumetinib versus Capecitabin	38	5.3 vs. 4.9

**Table 5:** Ongoing active clinical trials in pancreatic cancer targeting PI3K*.* Adapted from clinical.gouv.fr, updated in April 2018. Most phase I clinical trials using PI3K inhibitors in monotherapy or in combination therapy are completed; they include advanced pancreatic cancers (ref pons-Tostivint).

Name of the Study	Molecule Tested	Type of Therapy	Type of Drug	Type of Patients	Phase
NCT03065062	Palbociclib + Gedatolisib	Targeted therapy	CDK4/6 inhibitor + PI3K/mTOR inhibitor	Solid tumors	I
NCT02646748	Pembrolizumab + Itacitinib (INCB039110) and/or Pembrolizumab + INCB050465	Targeted therapy	PD-1 antibody + JAK1 inhibitor and/or PD-1 antibody + PI3Kδ inhibitor	Solid tumors	I
MATCH Screening trial	Multiple (including GSK2636771)	Targeted therapy	Multiple (including PI3Kβ inhibitor)	Advanced refractory solid cancers	II
NCT02077933	Alpelisib + Everolimus or Alpelisib + Everolimus + Exemestane	Targeted therapy	PI3Kα inhibitor + mTOR inhibitor or PI3Kα inhibitor + mTOR inhibitor + aromatase inhibitor	Advanced breast, renal and pancreatic cancer	I
NCT02155088	BYL719 + Gemcitabine + Nab-Paclitaxel	Targeted therapy + chemotherapy	PI3Kα + Anti-metabolite + Microtubule poison	Locally advanced and metastatic pancreatic cancer	I

## Glossary

Targeted therapy: Also known as molecularly targeted therapy. In cancer, these therapies differ from conventional therapies as they act on specific molecular targets that aim to interfere with crucial cell processes. Precision medicine: Emerging medical field that compiles information coming from a patient’s genes and proteins to prevent, diagnose and/or treat disease. Heterotypic signalling: Signalling between different cell types. intrinsic resistance: Innate ability to show resistance or to tolerate a treatment. Adaptative resistance: Acquired resistance towards a treatment/molecule due to frequent exposure.

## Abbreviations

ALK	Anaplastic lymphoma kinase;
APOA2	Apolipoprotein A2;
APOA4	Apolipoprotein A4;
ASCO	American Society of Clinical Oncology;
BTK	Bruton’s tyrosine kinase;
CA 19-9	Cancer antigen 19-9;
CDK4/6	Cyclin-dependent kinase 4/6;
CSF-1R	Colony-stimulating factor 1 receptor;
CP	Chronic pancreatitis;
CTC	Circulating tumoral cell;
DDR1/2	Discoidin domain receptor tyrosine kinase 1/2;
EGFR	Epidermal growth factor receptor;
ELISA	Enzyme-linked immunosorbent assay;
FFPE	Formalin-fixed paraffin-embedded;
FGFR	Fibroblast growth factor receptor;
FLT3	Fms-related tyrosine kinase 3;
GAS6	Growth arrest-specific 6;
Grb10	Growth factor bound protein 10;
HER2	human epidermal growth factor receptor 2;
Hsp27	Heat shock protein 27;
IGF-1(R)	Insulin-like growth factor 1 (Receptor);
IMAC	immobilized metal affinity chromatography;
IPMN	Intraductal papillary mucinous neoplasm;
iTRAQ	isobaric tags for relative and absolute quantitation;
JAK	Janus kinase;
Lck	Lymphocyte-specific protein tyrosine kinase;
LCM	Laser capture microdissection;
MA	Marketing authorization;
MALDI-TOF	Matrix assisted laser desorption ionization-time of flight mass spectrometry;
MEK	Mitogen-activated protein kinase kinase;
MET	Hepatocyte growth factor receptor;
MRM	Multiple reaction monitoring;
MS	Mass spectrometry;
mTOR	Mammalian target of rapamycin;
NNRTI	Inhibitor of non-nucleoside reverse transcriptase;
PanIN	Pancreatic intraepithelial neoplasia;
PARP	Poly ADP ribose polymerase;
PDAC	Pancreatic ductal adenocarcinoma;
PDGFR	Platelet-derived growth factor receptor;
PDX	Patient-derived xenograft;
PI3K	Phosphatidylinositol-3-kinase;
PSCA	Prostate stem cell antigen;
pTyr	phosphorylated tyrosine;
OS	Overall survival;
PFS	Progression-free survival;
RANKL	Receptor activator of nuclear factor kappa-Β ligand;
ROS1	C-ros oncogene 1;
RPPA	Reverse phase protein array;
RTK	Receptor tyrosine kinase;
Tie2	Tyrosine kinase with immunoglobulin and EGF homology domains;
TIMP	Tissue inhibitor of metalloproteinase-1;
TiO_2_	Titanium dioxide;
TGFβR	Transforming growth factor β receptor;
TK	Tyrosine kinase;
SILAC	Stable isotope labelling with amino acids in cell culture;
SMO	Smoothened kinase;
SRM	Selected reaction monitoring
VEGF(R)	Vascular endothelial growth factor (receptor);
